# Long non-coding RNA MIR100HG promotes the migration, invasion and proliferation of triple-negative breast cancer cells by targeting the miR-5590-3p/OTX1 axis

**DOI:** 10.1186/s12935-020-01580-6

**Published:** 2020-10-16

**Authors:** Fei-Yu Chen, Zhi-Yang Zhou, Ke-Jing Zhang, Jian Pang, Shou-Man Wang

**Affiliations:** 1 Department of Breast Surgery, Xiangya Hospital, Central South University, No. 87 Xiangya Road, Changsha, 410008 Hunan People’s Republic of China; 2 Clinical Research Center For Breast Cancer In Hunan Province, Changsha, 410008 Hunan People’s Republic of China

**Keywords:** TNBC, MIR100HG, miR-5590-3p, OTX1

## Abstract

**Background:**

As an aggressive subtype of breast cancer with a high risk of recurrence, triple-negative breast cancer (TNBC) lacks available treatment targets. LncRNA MIR100HG promotes cell proliferation in TNBC. However, few studies have investigated the molecular mechanism of MIR100HG in TNBC. Thus, additional in-depth investigations are needed to unravel its associated regulatory mechanism.

**Methods:**

MIR100HG and miR-5590-3p expression in TNBC tissue samples and cell lines was detected by RT-qPCR. Flow cytometry, transwell, wound-healing, CCK8 and colony formation assays were performed to analyse cell apoptosis, cell cycle, invasion, migration and proliferation. The protein expression of orthodenticle homeobox 1 (OTX1) and proteins in the ERK/MAPK signalling pathway were assessed by western blot analysis. Bioinformatics and luciferase assay were performed to predict and validate the interaction between MIR100HG and miR-5590-3p as well as OTX1 and miR-5590-3p. RNA immunoprecipitation (RIP) was used to detect the interaction between MIR100HG and miR-5590-3p. Subcutaneous tumour growth was observed in nude mice. Immunohistochemistry (IHC) analysis was used to assess OTX1 expression in tumour tissues.

**Results:**

MIR100HG expression was upregulated, whereas that of miR-5590-3p was downregulated in TNBC. MIR100HG was shown to directly interact with miR-5590-3p. Furthermore, MIR100HG knockdown could promote TNBC cell apoptosis and cell cycle arrest in G0/G1 phase while inhibiting migration, invasion and proliferation. Furthermore, miR-5590-3p inhibition showed the opposite results and could reverse the effect of MIR100HG knockdown in TNBC cells. MiR-5590-3p downregulated the ERK/MAPK signalling pathway, suppressed the migration, invasion and proliferation of TNBC cells and promoted their apoptosis and cell cycle arrest in G0/G1 phase by targeting OTX1. In addition, MIR100HG knockdown inhibited OTX1 expression by upregulating miR-5590-3p in vivo, thereby inhibiting tumour growth.

**Conclusions:**

MIR100HG promotes the progression of TNBC by sponging miR-5590-3p, thereby upregulating OTX1, suggesting a new potential treatment target for TNBC.

## Background

As the most common malignancy in women, breast cancer has become the dominant cause of cancer-associated death and is divided into a variety of molecular subtypes, among which TNBC is the most aggressive and has a high risk of recurrence [1, 2]. Due to the absence of progesterone receptor (PR), oestrogen receptor (ER) and epidermal growth factor receptor 2 (HER-2) being characteristics of TNBC, effective targeted therapies remain lacking for this disease [3], with the 5-year survival rate of patients being only 60% [4]. Thus, it is of great importance to elucidate the molecular mechanisms associated with the development of TNBC, which will pave the way to develop novel effective therapies for this malignancy.

LncRNAs are a class of noncoding RNAs longer than 200 nucleotides without coding potential that are involved in the regulation of various diseases, including cancer [5, 6]. Recently, lncRNAs have been recognized to play important roles in the genesis and progression of TNBC [7–9]. However, the underlying molecular mechanisms associated with this process require further elucidation. Some lncRNAs are referred to as miRNA-host gene lncRNAs (lnc-miRHGs), as they harbour miRNAs within their sequences [10]. However, only a few studies to date have focused on the miRNA-independent roles of lnc-miRHGs that are independent of the miRNAs from which they are processed. LncRNA MIR100HG takes part in cancer progression in both miRNA-independent and -dependent manners. For example, it can promote the migration and proliferation of laryngeal squamous cell carcinoma cells [11] and can also function as oncogene in acute megakaryoblastic leukaemia [12]. Recently, the regulatory role of MIR100HG in promoting TNBC cell proliferation has also been reported [13]. However, research on the molecular mechanism of MIR100HG in TNBC is currently limited. Thus, additional efforts should be made to unravel its regulatory mechanism in greater depth.

MicroRNAs (miRNAs) are approximately 20-22nt long endogenous RNAs that are involved in regulating multiple physiological biological processes, such as tumourigenesis and metastasis [14]. MiRNAs are also involved in the progression of TNBC [15]. Recent studies have shown that miR-5590-3p is also involved in the regulation of cancer, being able to inhibit diffuse large B cell lymphoma progression and immune evasion [16]. In addition, it can also regulate tumour growth and metastasis in hepatocellular carcinoma through the Wnt/β-catenin pathway [17]. In particular, miR-5590-3p is downregulated in TNBC [18]. However, the relationship between miR-5590-3p and MIR100HG has not been reported. Thus, it is of great importance to investigate the regulatory mechanism between miR-5590-3p on MIR100HG in TNBC progression.

In the present study, we assessed biological function of MIR100HG in TNBC and present evidence that it promotes tumourigenesis in TNBC through the miR-5590-3p/OTX1 axis. Collectively, the results of the present study elucidated the molecular mechanism of MIR100HG in regulating TNBC progression and explored the potential of MIR100HG inhibition as an anti-TNBC therapeutic target.

## Methods

### Patient tissues

The present study was approved by the Ethics Committee of Xiangya Hospital of Central South University. The study followed the tenets of the Declaration of Helsinki, and written informed consent was obtained from all patients. Twenty paired TNBC tissue samples and adjacent normal tissue samples were collected during surgical resection of breast cancer from patients at Xiangya Hospital of Central South University. Specimens were immediately frozen in liquid nitrogen after surgery.

### Cell lines

The cell line HEK-293T, the normal human breast epithelial cell line MCF-10A and the TNBC cell lines (MDA-MB-231, MDA-MB-453, MDA-MB-468 and MDA-MB-415) were all purchased from the American Type Culture Collection (ATCC, USA). HEK-293T cells were cultured in Dulbecco’s modified Eagle’s medium (DMEM; Invitrogen, USA) supplemented with 10% foetal bovine serum (FBS; Invitrogen, USA). MCF10A and TNBC cells were cultured in DMEM/F12 (Invitrogen, USA) supplemented with 5% horse serum (Thermo Fisher, USA), 0.5 µg/ml of hydrocortisone, 20 ng/ml of epidermal growth factor, 10 µg/ml of insulin and 100 ng/ml of cholera toxin. All cells were cultured under a humidified atmosphere with 5% CO_2_ at 37℃.

### Cell transfection

To overexpress OTX1, the full-length OTX1 cDNA sequence was amplified and inserted into the vector pcDNA3.1 (Life Technologies, San Francisco CA, US). The resulting vector (OE-OTX1) was used to overexpress OTX1, and empty pcDNA3.1 served as a control. MIR100HG and the negative control (NC) shRNAs (2 µg, Genepharma, China) were used in gene silencing assays. MiR-5590-3p mimics, the miR-5590-3p inhibitor and their NCs were purchased from GenePharma. MIR100HG shRNA (sh-MIR100HG) plasmids, OE-OTX1, miR-5590-3p mimics and the inhibitor as well as their negative controls were transiently transfected into cells with Lipofectamine 3000 transfection reagent (Invitrogen, USA) 24 h after being seeded. Then, 48 h later, the cells were harvested for subsequent assays.

### Cell viability assays

A Cell Counting Kit-8 (CCK8) kit (DOJINDO, Japan) was used to test cell viability according to the manufacturer’s instructions. Cells were cultured and transfected in 96-well plates. Cell proliferation was recorded every 24 h after being transfected. The optical density (OD) of samples was assessed at 450 nm with a spectrophotometer (Thermo Fisher, USA). All experiments were performed in triplicate.

### Luciferase reporter assay

MIR100HG sequence fragments and the 3’-untranslated region (3’-UTR) of OTX1 containing predicted miR-5590-3p binding sites and their mutant sequences were sub-cloned into the plasmid pmirGLO (Promega, USA) to generate MIR100HG wild-type (MIR100HG-WT), OTX1 wild-type (OTX1-WT), MIR100HG mutant (MIR100HG-MUT) and OTX1 mutant (OTX1-MUT) constructs. MiR-5590-3 mimics or inhibitors (50 pmol/well) and luciferase reporter plasmids (800 ng/well) were co-transfected into HEK-293T cells with Lipofectamine 3000 (Invitrogen, USA). Then, the relative luciferase activities were measured with a dual-luciferase assay system (Promega, USA) 48 h after transfection.

### RNA immunoprecipitation (RIP) assays

An EZMagna RIP kit (Millipore, USA) was used to perform RIP analysis following the manufacturer’s protocol. Briefly, 10 µL (10%) cell lysate supernatant was used as input. The rest of cell lysates were incubated with specific antibodies (anti-Ago2, 1:1 000 dilution and IgG as control) (Abcam, MA, USA) overnight at 4 ℃, followed by pulling down with protein G Sepharose 4 Fast Flow suspension (GE Amersham, Little Chalfont, UK). The beads were washed with lysis buffer and digested with proteinase K (Sangon, Shanghai, China) for 1 h. The elutes were used for RNA purification with Trizol reagent (Invitrogen, Missouri, USA), RT-qPCR was performed to analyse the levels of specific RNA transcripts.

### Wound-healing assay

Sterile 200-µl pipette tips were used to scratch cells to create a wound after they had grown to 80% confluence in a 6-well plate. After washing and removing floating cells with PBS, the cells cultured in medium without serum. The cells were imaged at 0 and 24 h, and NIS-Elements AR 3.1 (Nikon, Japan) was used to quantify the total distance travelled by the cells.

### Transwell assay

Transwell permeable supports (24 well, 8 µm pore size, Corning, USA) were used to measure the invasion capability of TNBC cells. After the upper chamber was coated at 37℃ with 200 µl of diluted BD Matrigel™ Basement membrane Matrix (BD, USA) gel solution for 4 h, 800 µl DMEM supplemented with 10% FBS was added to the lower chamber. Then, the upper chamber was seeded with 1 × 10^4^ cells in serum-free medium, and the cells were incubated at 37℃ for 24 h. Subsequently, the cells were fixed in 4% paraformaldehyde and stained with 1% crystal violet, after which cotton swabs were used to gently remove the cells remaining on the upper membrane. We counted stained cells under a microscope and assessed cell invasion from five random fields.

### Colony formation assay

For colony formation assays, 2 × 10^3^ cells were seeded in 6-well plates and incubated for 2 weeks. Then, 4% paraformaldehyde was used to fix the colonies, after which they were stained with 1% crystal violet (Sigma-Aldrich, USA). The numbers of visible colony were scored and analysed.

### Flow cytometry analysis

Flow cytometry was used to analyse cell cycle and cell apoptosis. Briefly, for cell cycle analysis, a Cell Cycle Analysis kit (GenScript, China) was used, where cells were collected and fixed in 70% ethanol for 2 h at 4℃ before being stained with RNase and PI reagent in darkness according to the manufacturer’s instructions. For the apoptosis assay, an Annexin V-FITC Apoptosis Detection kit (KeyGen Biotech, China) was used, where the cells were stained with propidium iodide (PI) and FITC. Then, a FACScan system (BD Biosciences, USA) was used to assess all samples.

### Xenograft mouse models

The animal experiments were approved by the Animal Care and Use Committee of Xiangya Hospital of Central South University. All animal experiments were conducted according to the Guideline for the Care and Use of Laboratory Animals. Eighteen six-week-old male BALB/c athymic nude mice were purchased from the National Laboratory Animal Center (Beijing, China) and were randomized and equally assigned into control, sh-NC or sh-MIR100HG groups (n = 05). Subsequently, 1 × 10^6^ MDA-MB-231 cells transfected with sh-NC or sh-MIR100HG were injected subcutaneously into the left flank. Then, on the 3rd, 7th, 14th, 21st and 28th days, 3 mice were randomly sacrificed to collect tumour tissues and measured the sizes and weights of the tumours. Tumour volumes were determined according to the equation: 0.5 × length × width^2^. Twenty-eight days after tumour cell injection, the mice were killed by cervical dislocation, and the tumour tissues were then weighed and carefully preserved.

### Immunohistochemistry (IHC) analysis

Briefly, tissues were collected, fixed in neutral formalin, dehydrated, and then embedded in paraffin. Then, the tissues were sliced into serial sections (5-µm thick), dehydrated and then incubated at room temperature (RT) in 3% hydrogen peroxide for 15 min before performing high-temperature antigen retrieval. Then, the sections were blocked with normal nonimmune animal serum and allowed to stand at RT for 20 min, after which they were treated with the primary antibody rabbit anti-OTX1 (1:200, Abcam, USA) and incubated at 4℃ overnight. Subsequently, the samples were treated with a biotinylated goat anti-rabbit IgG secondary antibody and incubated at 37℃ for 20 min. Adiaminobenzidine chromogen (DAB) solution was used to develop the colour reaction. The sections were then treated with haematoxylin, dehydrated, permeabilized, and mounted. Finally, the samples were observed under a microscope (Nikon, Japan).

### Quantitative reverse transcription PCR (RT-qPCR)

Total RNA was extracted with TRIzol regent (Invitrogen, USA). Then, first-strand cDNA was generated using an ImProm-II Reverse Transcription System (Promega, USA). KOD SYBR® qPCR Mix (Toyobo, China) was used for qPCR analysis. The following primer sequences were used: MIR100HG forward: 5′-CAC TGG TCT GCC CTT CCT AA-3′ and reverse: 5′-GGG GAT GAA CCA TTG ACA AC-3′; OTX1 forward: 5′-CTG CTC TTC CTC AAT CAA TGG-3’ and reverse: 5′-ACC CTG ACT TGT CTG TTT CC-3′; miR-5590-3p forward: 5′-GCG GCG AAT AAA GTT CAT GTA-3′ and reverse: 5′-GTC GTA TCC AGT GCA GGG TCC GAG GTA TTC GCA CTG GAT ACG ACT TGC CA-3′; U6 forward: 5′-CTC GCT TCG GCA GCA CA-3′ and reverse: 5′-AAC GCT TCA CGA ATT TGC GT-3′; and GAPDH forward: 5’-CCA GGT GGT CTC CTC TGA-3’ and reverse: 5’- GCT GTA GCC AAA TCG TTG T-3′.

### Western blot analysis

Protein lysis buffer (Solarbio, China) containing protease inhibitor cocktail (Roche, Switzerland) was used to isolate total protein. Then, protein samples were separated by 10% SDS-PAGE and transferred to nitrocellulose membranes (Millipore, USA). Then, the membranes were probed with specific primary antibodies against the following proteins: β-actin(CST, USA, 1:5000), OTX1 (CST, USA, 1:1000), MEK (CST, USA, 1:1000), p-MEK (CST, USA, 1:1000), ERK (CST, USA, 1:1000), p-ERK (CST, USA, 1:1000), MAPK (CST, USA, 1:1000), p-MAPK (CST, USA, 1:1000), JNK (CST, USA, 1:1000), and p-JNK (CST, USA, 1:1000). Subsequently, the membranes were incubated with a secondary antibody (goat anti-rabbit, Abcam, 1:5000) at RT for 1 h. Protein bands were then visualized with a Molecular Imager Gel Doc XR system (Bio-Rad, USA) and analysed with Photoshop (Adobe, USA).

### Statistical analysis

All experiments were repeated at least three times. Statistical analyses were conducted using SPSS 21.0 (IBM, USA). The data are presented as the means ± standard deviation (SD). One-way analysis of variance (ANOVA) was used for comparisons between multiple groups, while unpaired Student’s t-test (two-tailed) was used for two groups. P < 0.05 was considered statistically significant.

## Results

### MIR100HG and miR-5590-3p are differentially expressed in TNBC tissues and cells

**Fig. 1:**
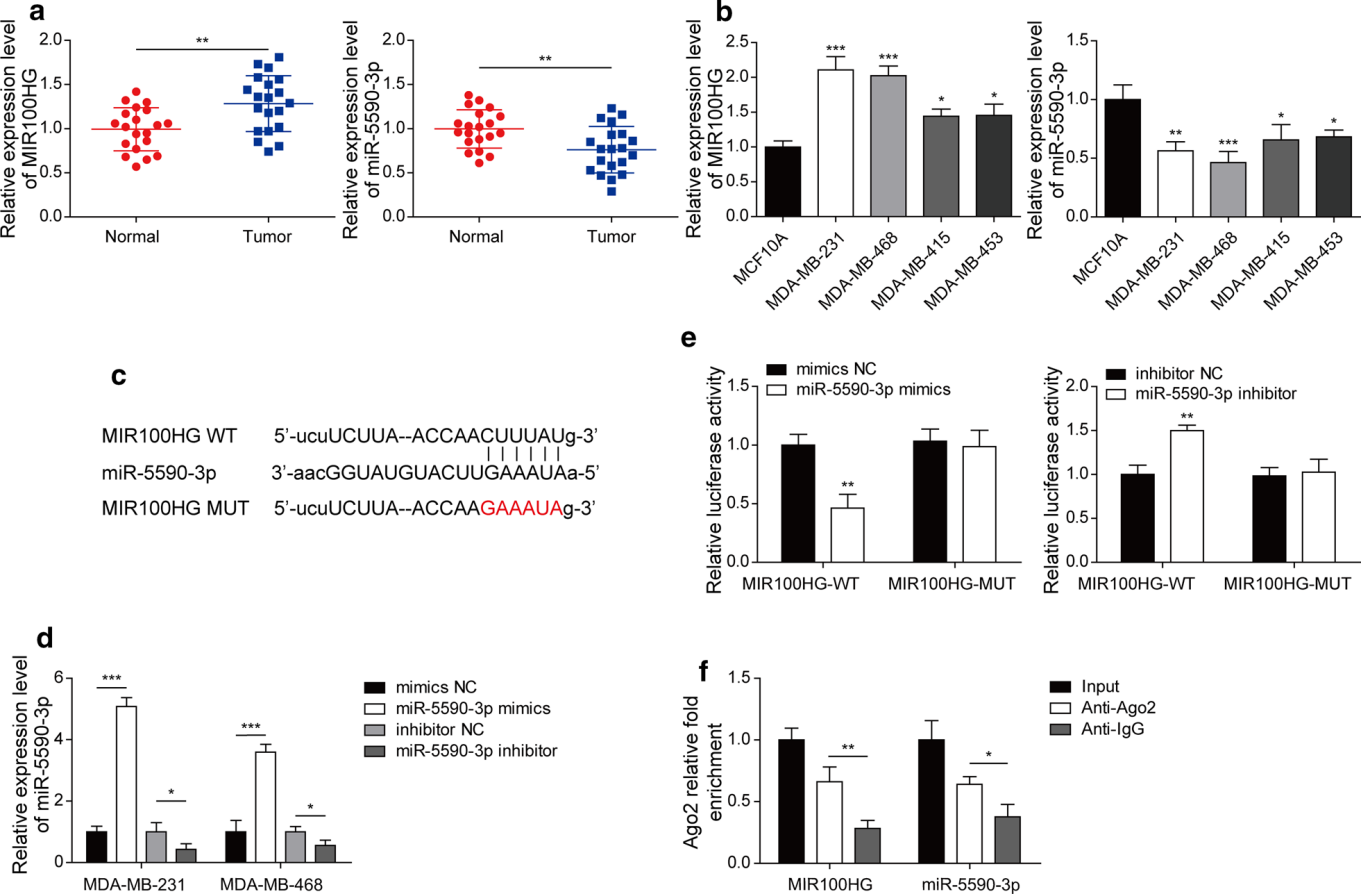
MIR100HG and miR-5590-3p expression patterns in TNBC cells and tissues and demonstration that MIR100HG directly targets miR-5590-3p. **a** The relative expression of MIR100HG and miR-5590-3p in TNBC tissues and their paired non-cancerous tissues (N = 20), as detected by RT-qPCR. **b** The expression level of MIR100HG and miR-5590-3p expression in TNBC and human breast epithelial cell lines, as detected by RT-qPCR. **c** The predicted miR-5590-3p binding site on MIR100HG. **d** MiR-5590-3p expression in TNBC cells after being transfected with the miR-5590-3p mimics and inhibitor. **e** The luciferase activity of MIR100HG-WT and MIR100HG-MUT in HEK-293T cells transfected with the miR-5590-3p mimics and inhibitor. **f** Relative expression level of MIR100HG and miR-5590-3p following Ago2 immunoprecipitation or IgG control immunoprecipitation.*P < 0.05, **P < 0.01, ***P < 0.001

MIR100HG and miR-5590-3p in 20 pairs of TNBC and adjacent normal tissues was assessed by RT-qPCR (Fig. 1a). Increased MIR100HG expression was observed in TNBC tissues (Fig. 1a) and MDA-MB-231, MDA-MB-453, MDA-MB-415 and MDA-MB-468 cell lines, whereas that of miR-5590-3p was downregulated (Fig. 1b). These results indicated a negative correlation between MIR100HG and miR-5590-3p expression in TNBC tissues and cells.

### MIR100HG can directly target miR-5590-3p

Subsequently, the ability of MIR100HG to bind miR-5590-3p was investigated. The potential miR-5590-3p binding sites in MIR100HG were predicted through bioinformatics analysis (Fig. 1c). MiR-5590-3p mimics and an inhibitor as well as their negative controls were transfected into MDA-MB-468 and MDA-MB-231 cells. The results presented in Fig. 1d show that the miR-5590-3p mimics successfully increased miR-5590-3p expression, while the miR-5590-3p inhibitor downregulated miR-5590-3p expression. Luciferase reporters wild-type MIR100HG (MIR100HG-WT) and its mutated form without the miR-5590-3p binding site (MIR100HG-MUT) were also constructed. The miR-5590-3p mimics significantly inhibited the luciferase activity of the MIR100HG-WT reporter but not that of the mutant construct in HEK-293 cells, while the MIR100HG-WT but not the MIR100HG-MUT reporter exhibited increased activity when cells were treated with the miR-5590-3p inhibitor (Fig. 1e). As the ability to bind the protein Ago2 is a distinct feature of competing endogenous RNAs (ceRNAs), whether MIR100HG interacted with Ago2 was also assessed through RIP analysis. As shown in Fig. 1f, MIR100HG and miR-5590-3p were both significantly enriched in Ago2-containing miRNA ribonucleoprotein complexes relative to the control group (anti-IgG group). Taken together, these results indicate that MIR100HG can directly target miR-5590-3p.

### MIR100HG knockdown inhibits the invasion and migration of TNBC cells by upregulating miR-5590-3p expression

**Fig. 2:**
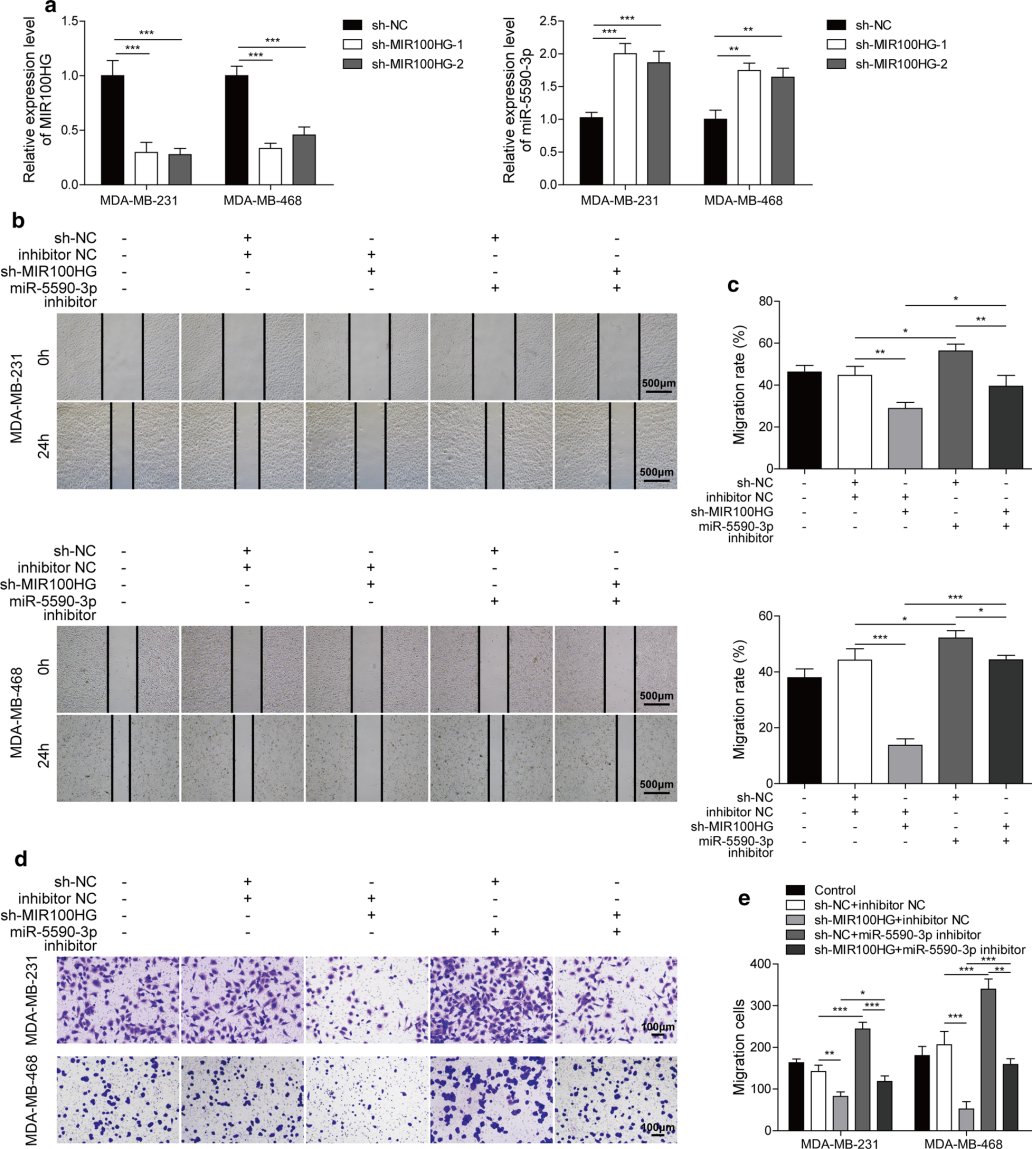
MIR100HG knockdown inhibits the invasion and migration of TNBC cells by regulating miR-5590-3p expression.**a** The relative expression of MIR100HG and miR-5590-3p in TNBC cells after MIR100HG knockdown. **b**, **c** The migration of TNBC cells, as assessed by wound healing assays. Scale bar: 500 µm. **d**, **e** The invasive capacity of TNBC cells, as assessed by transwell assays. Scale bar: 100 µm. *P < 0.05, **P < 0.01, ***P < 0.001

Next, we investigated the molecular mechanism by which MIR100HG regulates the migration and invasion of TNBC cells. MDA-MB-231 and MDA-MB-468 cells were used for further investigation because of their high level of MIR100HG expression and were transfected with sh-MIR100HG or sh-NC. As shown in Fig. 2a, miR-5590-3p expression was upregulated while that of MIR100HG was significantly downregulated by sh-MIR100HG. Sh-MIR100HG-1 was more effective in knocking down MIR100HG and was therefore chosen for further investigation. Then, migration assay results showed that TNBC cell migration was inhibited by MIR100HG knockdown, whereas miR-5590-3p inhibition promoted TNBC cell migration and could reverse the inhibition of TNBC cell migration mediated by MIR100HG knockdown (Fig. 2b and c). In addition, MIR100HG knockdown also inhibited TNBC cell invasion. Furthermore, miR-5590-3p inhibition promoted the invasive ability of TNBC cells and could compensate for the inhibition of TNBC cell invasion mediated by MIR100HG knockdown (Fig. 2d and e). Taken together, these results demonstrate that MIR100HG knockdown inhibited the invasion and migration of TNBC cells through miR-5590-3p.

### MIR100HG knockdown inhibits the proliferation and promotes the apoptosis of TNBC cells by upregulating miR-5590-3p expression

.

**Fig. 3:**
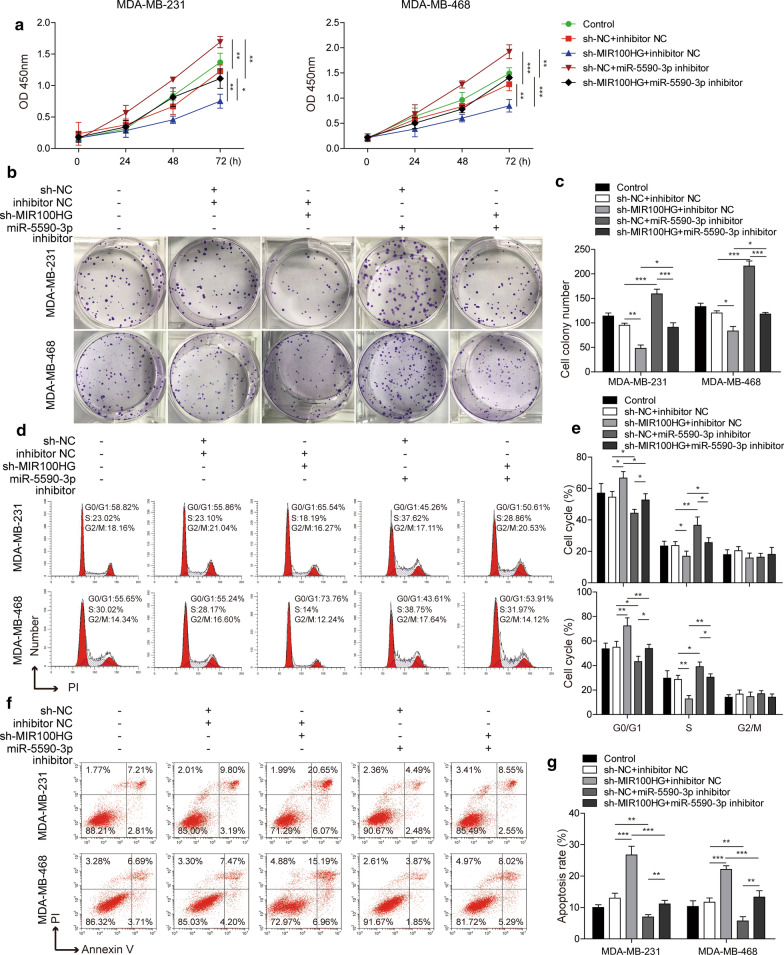
MIR100HG knockdown inhibits the proliferation and promotes the apoptosis of TNBC cells by regulating miR-5590-3p expression. **a** TNBC cell viability, as assessed by CCK8 assays. **b**, **c** TNBC cells proliferation, as assessed by colony formation assays. **d**, **e** Cell cycle analysis was performed by PI staining and subsequent flow cytometry. **f**, **g** TNBC cell apoptosis, as assessed by Annexin V/PI staining and subsequent flow cytometry analysis. *P < 0.05, **P < 0.01, ***P < 0.001

Next, the effect of MIR100HG on cell proliferation and apoptosis was further investigated. As shown in Fig. 3a, MIR100HG knockdown inhibited cell viability, while miR-5590-3p inhibition could promote cell viability and reverse the decrease in cell viability caused by MIR100HG knockdown. The results presented in Fig. 3b and c showed that MIR100HG knockdown inhibited TNBC cell proliferation.MiR-5590-3p inhibition could promote TNBC cell proliferation and reverse the suppression of cell proliferation mediated by MIR100HG knockdown. In addition, after MIR100HG knockdown, the number of G0/G1 and S phases increased and decreased, respectively. In contrast, after miR-5590-3p inhibition, the number of G0/G1 and S phase cells decreased and increased, respectively. Moreover, the miR-5590-3p inhibitor could reverse the effect of sh-MIR100HG(Fig. 3d and e). The apoptosis rate of TNBC cells was promoted by MIR100HG knockdown, while inhibition of miR-5590-3p suppressed TNBC cell apoptosis and inhibited the effects of MIR100HG knockdown (Fig. 3f and g). Therefore, these results indicated that MIR100HG knockdown inhibited the proliferation and promoted the apoptosis of TNBC cells through miR-5590-3p.

### MiR-5590-3p downregulates the ERK/MAPK signalling pathway by inhibiting OTX1

**Fig. 4:**
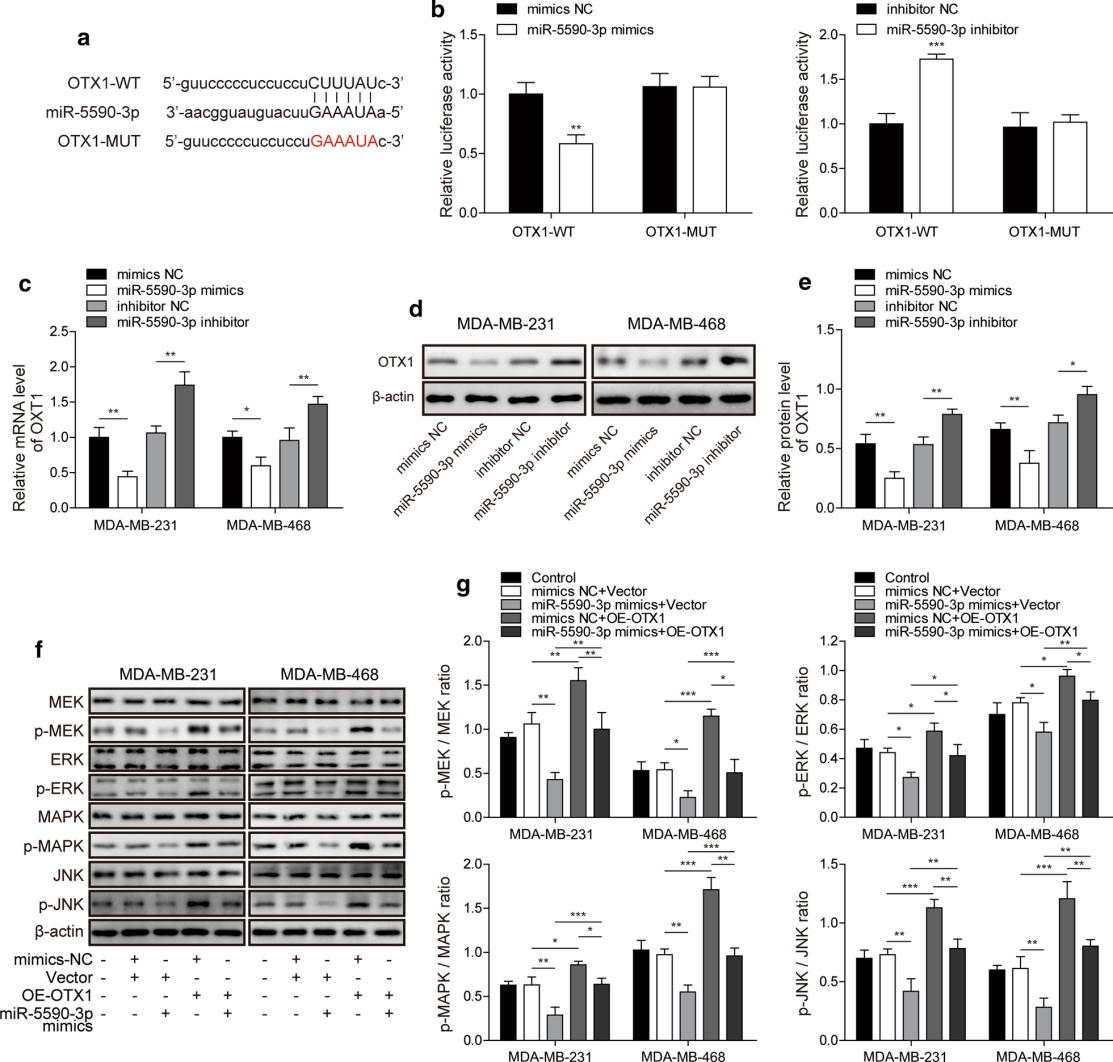
MiR-5590-3p downregulates the ERK/MAPK signalling pathway by targeting OTX1. **a** The predicted miR-5590-3p binding sites in OTX1. **b** The luciferase activity of OTX1-WT and OTX1-MUT reporter constructs in HEK293T cells transfected with the miR-5590-3p mimics and inhibitor. **c** The mRNA levels of OTX1 in TNBC cells transfected with the miR-5590-3p mimics and inhibitor, as detected by RT-qPCR. **d**, **e** The protein levels of OTX1 in TNBC cells transfected with the miR-5590-3p mimics and inhibitor, as detected by western blot analysis. **f**, **g** The protein level of ERK/MAPK signalling pathway after transfection, as detected by western blot analysis. *P < 0.05, **P < 0.01, ***P < 0.001

We subsequently attempted to elucidate the target of miR-5590-3p involved in regulating the progression of TNBC. A potential miR-5590-3p binding site in OTX1 mRNA was predicted through an in silico analysis (Fig. 4a). Then, luciferase reporters of OTX1 (OTX1-WT) and its mutated forms without miR-5590-3p binding site (OTX1-MUT) were also constructed. The results showed that the miR-5590-3p mimics and inhibitor significantly decreased and increased the luciferase activity of the OTX1-WT construct, respectively, while both treatments had no effect on the luciferase activity of mutant OTX1 construct (Fig. 4b). Furthermore, the miR-5590-3p mimics successfully downregulated OTX1 mRNA expression, while the miR-5590-3p inhibitor upregulated OTX1 mRNA expression (Fig. 4c). In addition, OTX1 protein levels were downregulated by miR-5590-3p mimics and upregulated by the miR-5590-3p inhibitor (Fig. 4d and e). Then, we assessed the expression of proteins in the ERK/MAPK signalling pathway. As shown in Fig. 4f and g, p-MEK, p-ERK, p-MAPK and p-JNK protein levels were all downregulated by treatment with the miR-5590-3p mimics, while OTX1 overexpression caused the opposite result and reversed the effect of the miR-5590-3p mimics. In addition, the total protein levels of these proteins were unaffected by OTX1 and miR-5590-3p overexpression. Thus, these results indicated that OTX1 is the downstream target of miR-5590-3p and that miR-5590-3p can downregulate the ERK/MAPK signalling pathway by targeting OTX1.

### MiR-5590-3p inhibits the migration and invasion of TNBC cells by inhibiting OTX1

**Fig. 5:**
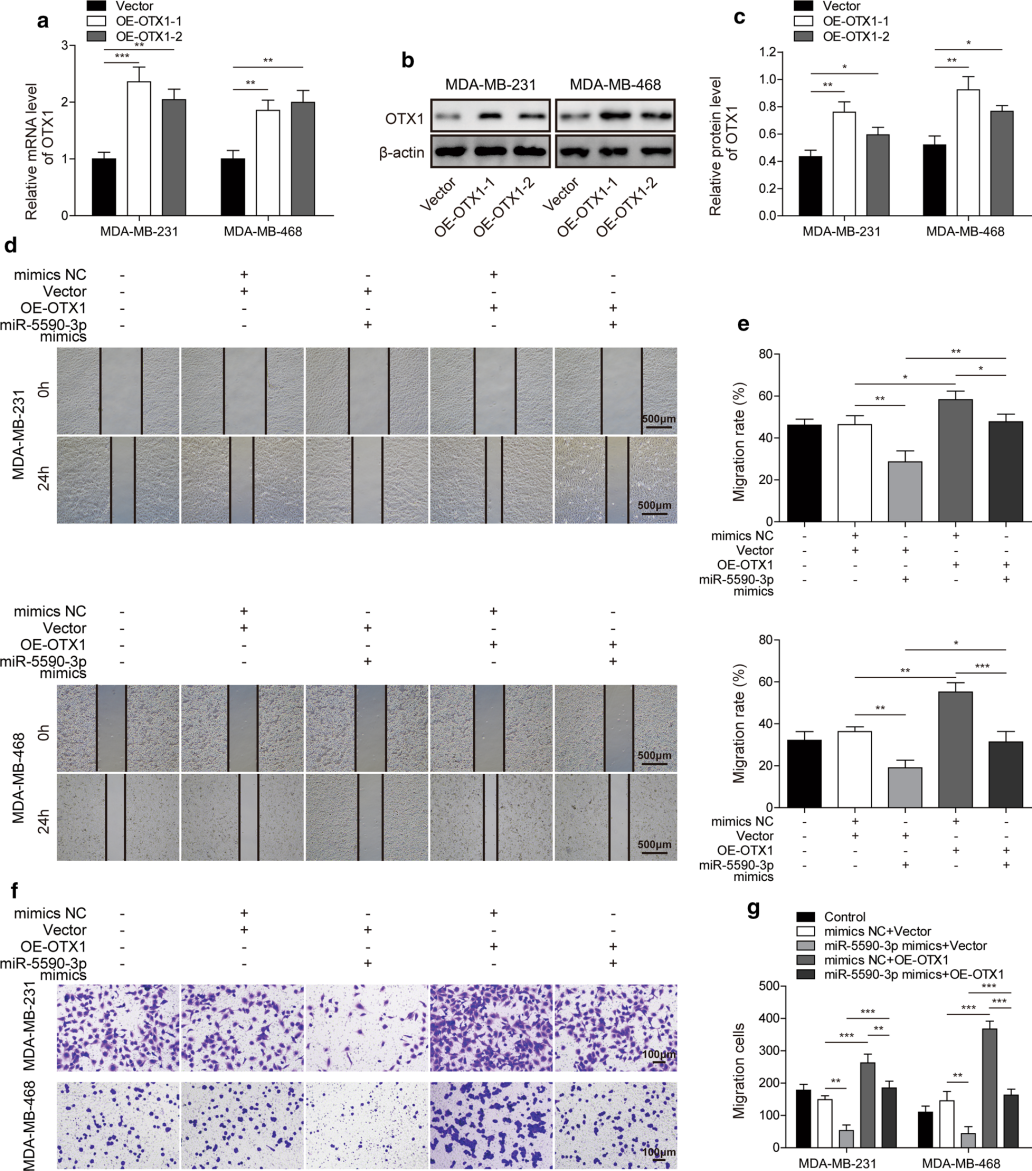
MiR-5590-3p inhibits the invasion and migration of TNBC cells through downregulation of OTX1 expression**a** The mRNA level of OTX1 in TNBC cells after OTX1 overexpression, as assessed by RT-qPCR. **b**, **c** The level of OTX1 protein expression in TNBC cells after OTX1 overexpression, as assessed by western blot analysis. C is a grayscale analysis chart of B western blot band. The protein level is normalized by β-actin, and then significant difference analysis is performed between OE-OTX1-1, OE-OTX1-2 and vector group. **d**, **e** The migration capacity of TNBC cells, as assessed by wound healing assays. Scale bar: 500 µm. **f**, **g** The invasive capacity of TNBC cells, as assessed by transwell assays. Scale bar: 100 µm. *P < 0.05, **P < 0.01, ***P < 0.001

Subsequently, whether binding between miR-5590-3p and OTX1 plays crucial roles in regulating the progression of TNBC was further investigated. The OTX1 overexpression vector was transfected into MDA-MB-468 and MDA-MB-231 cells. Both OTX1 mRNA (Fig. 5a) and protein (Fig. 5b and c) levels were successfully upregulated, and OE-OTX1-1 was used for subsequent experiments due to its higher overexpression. In migration assays, we observed that miR-5590-3p overexpression could inhibit TNBC cell migration, while OTX1 overexpression greatly enhanced TNBC cell migration and inhibited the effect of miR-5590-3p overexpression (Fig. 5d and e). Moreover, miR-5590-3p could also significantly inhibit the invasive ability of TNBC cells, while OTX1 overexpression greatly enhanced TNBC cell invasion and was able to inhibit the effect of miR-5590-3p (Fig. 5f and g). Therefore, these results indicated that miR-5590-3p can inhibit the migration and invasion abilities of TNBC cells by targeting OTX1.

### MiR-5590-3p inhibits the proliferation and promotes the apoptosis of TNBC cells by downregulating OTX1 expression

**Fig. 6:**
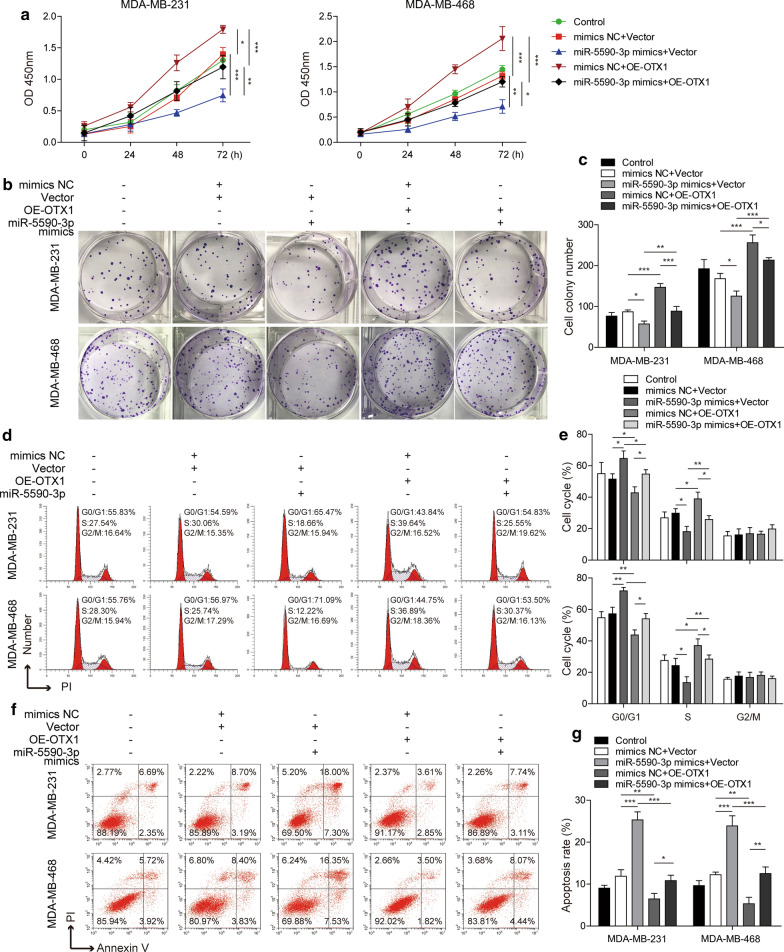
MiR-5590-3p inhibits the proliferation and promotes the apoptosis of TNBC cells by downregulating OTX1 expression**a** The viability of TNBC cells, as assessed by CCK8 assays. **b**, **c** The proliferation of TNBC cells, as assessed by colony formation assays. **d**, **e** Cell cycle analysis was performed by PI staining and subsequent flow cytometry. **f**, **g** The apoptosis of TNBC cells, as assessed by Annexin V/PI staining and subsequent flow cytometry analysis. *P < 0.05, **P < 0.01, ***P < 0.001

Subsequently, the effect of miR-5590-3p on TNBC cell apoptosis and proliferation was further investigated. The results presented in Fig. 6a show that miR-5590-3p overexpression inhibited cell viability, while OTX1 overexpression could promote cell viability and reverse the effect of miR-5590-3p overexpression. Similarly, miR-5590-3p inhibited TNBC cell proliferation, while OTX1 promoted TNBC cell proliferation and could inhibit the effect of miR-5590-3p overexpression (Fig. 6b and c). In addition, after miR-5590-3p overexpression, the numbers of G0/G1 and S phase cells increased and decreased, respectively, while after OTX1 overexpression, the number of cells in the G0/G1 phase decreased, while the number of cells in the S phase increased and reversed the effect of miR-5590-3p overexpression (Fig. 6d and e). In addition, miR-5590-3p overexpression also promoted the apoptosis of TNBC cells. Nevertheless, OTX1 overexpression suppressed TNBC cell apoptosis and compensated for the effect of miR-5590-3p overexpression (Fig. 6f and g). Therefore, our results indicated that miR-5590-3p inhibits the proliferation and promotes the apoptosis of TNBC cells by targeting OTX1.

### MIR100HG knockdown inhibits tumour growth via the miR-5590-3p/OTX1 axis in vivo

**Fig. 7:**
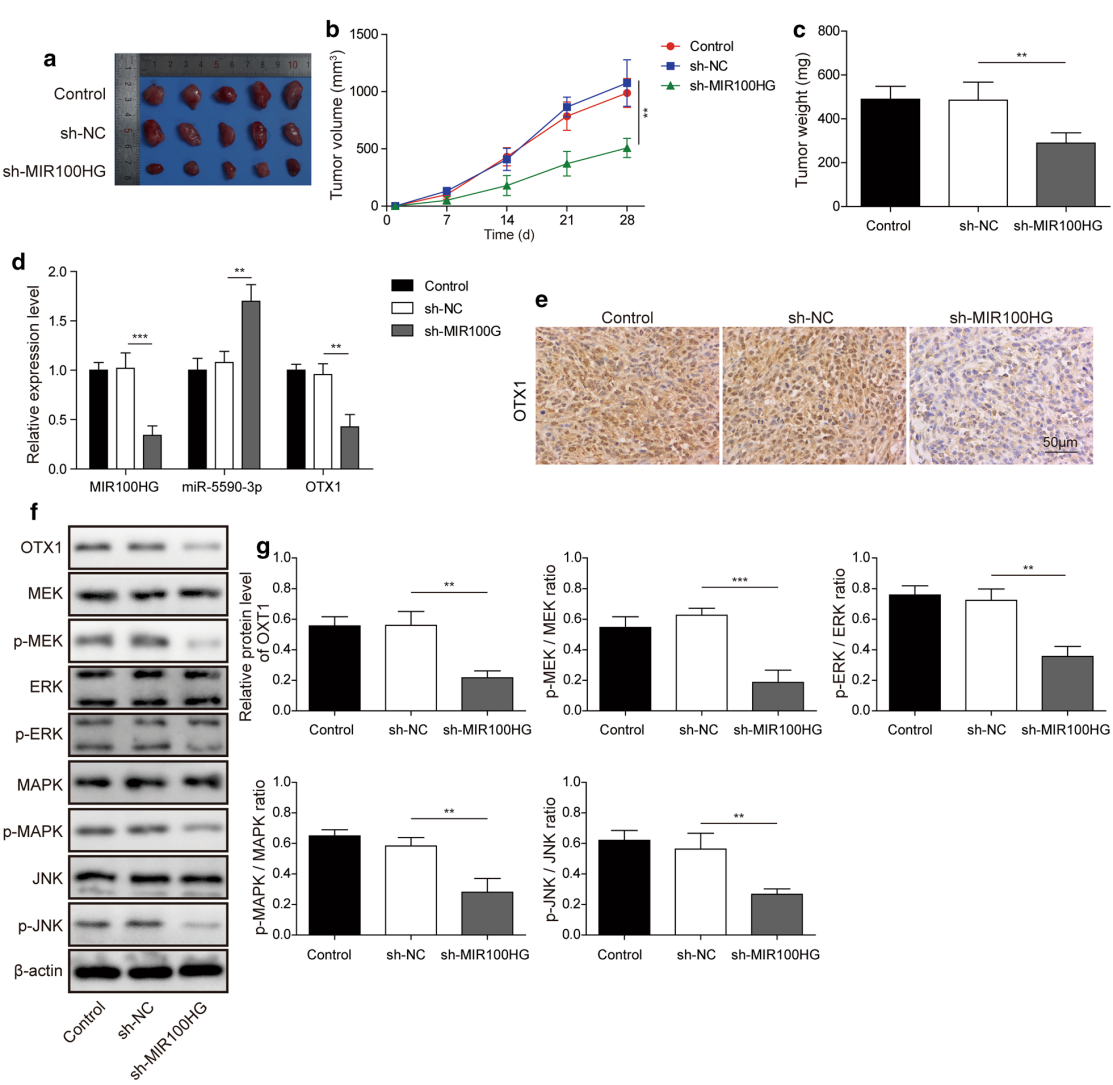
MIR100HG knockdown inhibits tumour growth by upregulating miR-5590-3p in a xenograft mouse model MDA-MB-231 cell xenograft tumours stably transfected with MIR100HG shRNA or shRNA Ctrl vectors were obtained. The sizes of the tumours was then recorded. **a**, **b** Periodic monitoring of tumour dimensions after MIR100HG knockdown. **c** Tumour weights on day 28. **d** The expression levels of MIR100HG, miR-5590-3p and OTX1 in tumour tissues on day 28. **e** The protein levels of OTX1 in tumour tissues, as assessed by IHC assays. Scale bar: 50 µm. **f**, **g** The protein levels in the ERK/MAPK signalling pathway and the OTX1 protein in tumour tissues, as assessed by western blot analysis. *P < 0.05, **P < 0.01, ***P < 0.001

To further evaluate the effect of MIR100HG knockdown on the development of TNBC, in vivo experiments in mice were performed. To evaluate the function of MIR100HG in tumour growth in vivo, we stably knocked down MIR100HG in MDA-MB-231 cells, which were then subcutaneously injected into nude mice. As shown in Fig. 7a and b, tumour volumes were smaller in the MIR100HG knockdown group compared with that observed in the control group. Consistent with these results, the average weight of tumours in the MIR100HG knockdown group was much lower than that of tumours in the control group (Fig. 7c). Then, we observed that MIR100HG and OTX1 expression were downregulated while that of miR-5590-3p was upregulated after MIR100HG knockdown (Fig. 7d). Moreover, OTX1 protein expression in different groups was further detected by IHC and western blot, and the results showed that OTX1 protein levels were downregulated after MIR100HG knockdown compared with that observed in the control group (Fig. 7e–g). In addition, phosphorylated MAPK, MEK, ERK and JNK protein levels were all downregulated after MIR100HG knockdown, while their total protein levels were unaffected (Fig. 7f and g). Taken together, these results suggest that MIR100HG knockdown inhibited tumour growth by increasing miR-5590-3p expression to inhibit the OTX1/ERK/MAPK pathway in vivo.

## Discussion

As TNBCs do not express the ER, HER-2 and PR genes, they do not respond to available targeted agents and endocrine therapy [3, 19], making it challenging to treat this malignancy. TNBC patients show a shorter median time to relapse and death [20]. Nevertheless, the molecular mechanism underlying the tumourigenesis and progression of TNBC is not fully understood and requires further investigation. In the present study, we demonstrated that MIR100HG was upregulated in TNBC cell lines and tissues and promoted the migration, invasion and proliferation of TNBC cells through the miR-5590-3p/OTX1 axis, providing a novel mechanistic role for MIR100HG in regulating TNBC progression that may be a potential treatment target for TNBC.

MiRNAs have been shown to have important functions in cancers, including TNBC. For example, miR-127 suppresses the proliferation and metastasis of TNBC cells [21]. In addition, miR-34a is involved in the regulation of EMT progression, cancer stemness and M2 macrophage polarization in TNBC [22], while miR-483-3p suppresses TNBC proliferation and progression by targeting HDAC8 [23]. Recently, miR-5590-3p has been reported to inhibit the proliferation and migration of TNBC cells [18]. However, its downstream molecular mechanism has not been fully elucidated. The results of the present study indicated that miR-5590-3p expression is reduced in TNBC tissues and cell lines and that miR-5590-3p overexpression can inhibit the growth of TNBC cell lines, while miR-5590-3p inhibition can promote TNBC cell growth.

OTX1is a homeobox gene that is a member of the OTX family and is important in the development of early human foetal retina and mammary gland [24]. In addition, OTX1 has recently been shown to be involved in the carcinogenesis of colorectal, bladder and hepatocellular cancer [25–27]. Nevertheless, whether this protein is involved in the carcinogenesis of TNBC is a target of miR-5590-3p have yet to be reported. In the present study, we demonstrated that OTX1 is a target of miR-5590-3p through dual-luciferase reporting assays and showed that it is negatively regulated by miR-5590-3p. OTX1 overexpression promoted the growth of TNBC cell lines and also inhibited the effects of miR-5590-3p overexpression. Thus, for the first time, we demonstrated that miR-5590-3p targets OTX1 and revealed the regulatory role of OTX1 in TNBC. Additionally, it was reported that miR-5590-3p inhibited tumour growth in gastric cancer by targeting DDX5 [28], and repressed diffuse large B cell lymphoma progression and immune evasion through targeting ZEB1 to regulate PD-1/PD-L1 checkpoint [29]. Whether miR-5590-3p also regulates cell function by targeting DDX5 or ZEB1 in TNBC deserves further study.

An increasing number of studies have revealed the regulatory functions of lncRNAs in the progression of cancer, including TNBC [30]. For example, lncRNABORG facilitates the survival and chemoresistance of TNBC [31], while lncRNA CCAT2 promotes TNBC oncogenesis by regulating the stemness of cancer cells [7]. In addition, LINC01638activates MTDH-Twist1 signalling in TNBC [32]. Recently, MIR100HG has been shown to promote TNBC cell proliferation through triplex formation with p27 loci [13]. In the present study, MIR100HG expression was shown to be significantly upregulated in both TNBC tissues and cells. MIR100HG knockdown could inhibit the growth of TNBC cells and tumours in vitro and in vivo. In addition, lncRNAs have been frequently reported to modulate the derepression of miRNA targets by acting as miRNA sponges and can take part in regulating cancer progression [33]. MIR100HGalso functions as a ceRNA and participates in the regulation of laryngeal squamous cell carcinoma progression by downregulating miR-204-5p [11]. However, there are no reports of MIR100HG regulating the progression of TNBC as a ceRNA. In the present study, we observed that miR-5590-3p inhibition could reverse the effect of MIR100HG inhibition in TNBC cell lines. MIR100HG was identified as a targeting miR-5590-3p, and its expression was negatively correlated with miR-5590-3p. Previous study demonstrated that lncRNA SNHG14 sponged miR-5590-3p to promote prostate cancer malignancy [34]. LncRNA SOX9-AS1 axis could play regulatory role in metastasis in hepatocellular carcinoma by targeting miR-5590-3p to regulate Wnt/β-catenin pathway [17]. SNHG14 and SOX9-AS1 in TNBC may also affect cell function by targeting miR-5590-3p. In addition, our results indicated that MIR100HG could upregulate OTX1 expression by sponging miR-5590-3p. These results indicated that the ceRNA regulation mode of MIR100HG plays a crucial role in the occurrence and development TNBC. To the best of our knowledge, we are the first to report this regulatory mechanism of MIR100HG in TNBC.

The ERK/MAPK signalling pathway, the most well-documented MAPK signalling pathway, is closely involved in cell proliferation and differentiation, angiogenesis and tumour metastasis [35], and it also plays a crucial role in the cell signal transduction network [36]. In the present study, MIR100HG knockdown successfully suppressed the activation of the ERK/MAPK signalling pathway in TNBC both in vitro and in vivo. To the best of our knowledge, this is a novel mechanism of MIR100HG in the oncogenesis in TNBC.

## Conclusions

In summary, the results of the present study provide the first clue of the role of MIR100HG as a ceRNA in the regulation of tumourigenesis and progression of TNBC and show, for the first time, that MIR100HG directly targets miR-5590-3p and upregulates OTX1 expression. These findings provide novel insights into the mechanisms underlying TNBC malignancy and suggests a new potential target to improve therapeutics for this disease in the future.

## Data Availability

Not applicable.

## Abbreviations

TNBC: Triple-negative breast cancer
lncRNAs: Long noncoding RNAs
OTX1: OrthodenticleHomeobox 1
IHC: Immunohistochemistry
RIP: RNA immunoprecipitation
ER: Oestrogen receptor
PR: Progesterone receptor
HER-2: Epidermal growth factor receptor 2
lnc-miRHGs: MiRNA-host gene lncRNAs
ceRNA: Competitive endogenous RNA
miRNAs: MicroRNAs
DMEM: Dulbecco’s modified Eagle’s medium
ATCC: American Type Culture Collection
FBS: Foetal bovine serum
OD: Optical density
PI: Propidium iodide
PBS: Phosphate-buffered saline
RT-qPCR: Quantitative reverse transcription PCR
RT: Temperature
DAB: Diaminobenzidine chromogen
SD: Standard deviation
